# Immunotherapy with adoptive cytomegalovirus‐specific T cells transfer: Summarizing latest gene engineering techniques

**DOI:** 10.1002/hsr2.322

**Published:** 2021-07-08

**Authors:** Mahshid Mehdizadeh, Samira Karami, Haniyeh Ghaffari Nazari, Ghazaleh Sankanian, Mohsen Hamidpour, Abbas Hajifathali

**Affiliations:** ^1^ Hematopoietic Stem Cell Research Center Shahid Beheshti University of Medical Sciences Tehran Iran

**Keywords:** adoptive T cell therapy, CAR T cell, CMV, stem cell transplantation, TCR‐engineered T cell, transgenic TCR

## Abstract

Cytomegalovirus (CMV) infection remains a major complication following allogeneic hematopoietic stem cell transplantation (HSCT). T cell response plays a critical role in inducing long‐term immunity against CMV infection/reactivation that impairs during HSCT. Adoptive T cell therapy (ACT) via transferring CMV‐specific T cells from a seropositive donor to the recipient can accelerate virus‐specific immune reconstitution. ACT, as an alternative approach, can restore protective antiviral T cell immunity in patients. Different manufacturing protocols have been introduced to isolate and expand specific T cells for the ACT clinical setting. Nevertheless, HLA restriction, long‐term manufacturing process, risk of alloreactivity, and CMV seropositive donor availability have limited ACT broad applicability. Genetic engineering has developed new strategies to produce TCR‐modified T cells for diagnosis, prevention, and treatment of infectious disease. In this review, we presented current strategies required for ACT in posttransplant CMV infection. We also introduced novel gene‐modified T cell discoveries in the context of ACT for CMV infection. It seems that these innovations are enabling to improvement and development of ACT utilization to combat posttransplant CMV infection.

## 1 INTRODUCTION

In the hematopoietic stem cell transplantation (HSCT) context, pre‐ and post‐HSCT processes such as conditioning regimens and graft‐vs‐host disease (GVHD) prophylaxis/treatment could weaken the immune system in transplanted patients. Furthermore, the long‐term immune reconstitution after transplantation increases the risk of viral infections, especially cytomegalovirus (CMV) reactivation in transplanted patients. Cell‐mediated immunity, which plays a vital role in controlling CMV infection, is impaired during the transplantation process.^1^ The majority of CMV‐specific cytotoxic T cells (CTLs) in patients are derived from donor cells during the early phase posttransplantation. However, thymus‐derived naïve T cells could recover after prolonged immune reconstitution and could be detected in the late phase posttransplantation. The expansion of CMV‐specific CTLs promotes an appropriate immune response in early and late posttransplantation phases.^2^ Although available anti‐CMV drugs have demonstrated desirable efficacy in preventing and treating posttransplant CMV infection, the risk of toxicity and resistance to these agents has limited their long‐term usage, leading to persistent and recurrent infections.^3^ Thus, there has always been a special requirement for developing safe, tolerable, feasible, and effective alternative therapies to overcome antiviral drug limitations. These alternative therapies include immunotherapeutic strategies which accelerate virus‐specific immune reconstitution and T cell recovery.^4^ In this regard, adoptive T cell therapy (ACT) through transferring viral‐specific T cells has been introduced as a rational approach to induce rapid and sufficient virus‐specific immunity in patients until reaching optimal immune reconstitution.^5^ Here we reviewed technical strategies of CMV‐specific T cell selection and isolation for treating CMV infection post HSCT and summarized new gene modifying‐based techniques to generate CMV‐specific T cells.

## 2 ADOPTIVE T CELL THERAPY

In the ACT, also called cellular adoptive immunotherapy, the donor's pathogen‐specific T cells are infused into a recipient and could be detected for a long time (up to 2 years) in vivo.^6^ Donor lymphocyte infusion (DLI), as a primary ACT method, is expected to contain memory T cells for a broad range of viruses and transfer antitumor and antiviral immunity from donors to recipients.^5^ Although DLI is an effective treatment approach for both viral infections and viral‐associated diseases, it could possibly increase the risk of GVHD^7^, ^8^, ^9^ and late CMV infection, especially in patients with early CMV infection history.^10^ Therefore, unmanipulated DLI administration is limited due to the probability of transferring alloreactive T cells (up to 10% of circulating T cells might be alloreactive) and low levels of antiviral‐specific T cells.^5^ Developing new methods in cell isolation, cell culture, and immunodominant epitope prediction has expanded virus‐specific T cell‐based adoptive therapy and facilitated ACT clinical use. Adoptive CMV‐specific T cell therapy has been explored for prophylactic and preemptive therapy following HSCT.^11^, ^12^, ^13^ In addition, it has become an interesting option to restore protective antiviral T cell immunity in CMV refractory/resistance allo‐HSCT patients.

## 3 CMV‐SPECIFIC ACT

Natural killer cells (NKG2C^+^ NK cells), CD16^+^ Vδ2^−^ γδ T cells, and conventional αβ CD8^+^ T cells are dominant immune cells in CMV‐infected immunocompetent patients.^14^ It has been reported that the frequency of CMV‐specific CD8^+^ T cells population may exceed 4% of the CD8^+^ T cell pool in CMV‐seropositive immunocompetent healthy donors.^15^ Therefore, CMV‐seropositive HLA‐matched donors are appropriate sources for immune‐based CMV therapy. Since naïve T cells have a broader T‐cell receptor (TCR) repertoire with an increased risk of alloreactivity, many pieces of research have been conducted based on transferring CMV‐specific memory T cells to restore virus‐specific immunity post‐HSCT.^16^, ^17^ Optimal CMV‐specific T cell therapy depends on several factors such as identification of CMV immunodominant antigens and HLA‐restricted epitopes, frequency of CMV‐specific T cell subsets in graft, and virus‐specific T cells isolation and enrichment techniques.

### 3.1 Characterization of immunodominant epitopes

Fortunately, CMV is an immunologically well‐characterized virus with immediate‐early 1 (IE1) protein and phosphoprotein of 65 kD (pp65) as major immunodominant antigens.^18^, ^19^ First adoptive CMV‐specific therapies were based on transferring major histocompatibility complex (MHC) class I‐restricted CD8^+^ T cell‐mediated immunity. Declining cytotoxic activity in patients with CMV‐specific CD4^+^ T cell deficiency suggests that CMV‐specific CD4^+^ T cells are required to exert and maintain CMV‐specific CD8^+^ T cells' antiviral effects [19–21]. Therefore, further adoptive immunotherapies were conducted by the infusion of products containing both CMV‐specific CD4^+^ and CD8^+^ T cell clones which are restricted to MHC‐II and MHC‐I, respectively.^20^ Subsequent studies declared that peripheral blood of healthy CMV seropositive adults contains both CD4^+^ and CD8^+^ CMV‐specific memory T cells targeting broad CMV genome epitopes, especially pp65 and IE.^21^, ^22^ Therefore, both MHC class I‐ and II‐restricted epitopes are essential to induce and maintain an optimal antiviral response. Advances in bioinformatics methods and cloning techniques facilitated the identification of overlapping peptide pools and viral vectors containing chimeric proteins, leading to the introduction of common (typical) and less‐common (atypical) epitopes that could be targeted by CMV‐specific CD4^+^/ CD8^+^ T cells.^21^, ^23^, ^24^ These epitopes are essential for CMV‐specific T cell activation and expansion. Efficient enrichment of both CD8^+^ and CD4^+^ CMV‐specific T cells has been reported following short‐time stimulation of donor peripheral blood cells with CMV pp65 and IE1 peptide pools.^25^ For example, HLA‐A*02‐restricted NLV (pp65 495‐503), HLA‐B*07‐restricted TPR (pp65 417‐426), HLA‐ A*01‐restricted YSE (pp65 363‐373), and HLA‐B*08‐restricted ELR (IE1199‐207) peptides are major CMV epitopes mostly used alone and in combined forms for ex vivo T cell expansion in adoptive CMV immunotherapy.^23^, ^26^, ^27^

### 3.2 Immunologic composition of the graft

CMV‐specific T cell subsets in graft can affect the success rate of CMV‐specific T cell adoptive therapy. Functional heterogeneity of CMV‐specific T cells derived from seropositive healthy donors has been shown in experimental studies.^15^ The differentiation of naïve and memory T cell subsets is according to the specific homing markers such as CD62L and CD45RO/RA. Naïve T cells are CD45RO^−^/CD45RA^+^/CD62L^+^, whereas central memory T cells (T_CM_) are CD45RO^+^/CD45RA^−^/CD62L^+^. Besides, effector memory T cells (T_EM_) are CD45RO^+^/CD45RA^−^/CD62L^−^ and terminally differentiated effector T cells (T_EMRA_) are CD45RO^−^/CD62L^−.^ ^28^ These immune cell subsets have various frequencies in the graft, affecting the prevention or treatment of CMV reactivation post‐HSCT. A study suggested that donor grafts containing a high frequency of CMV‐specific memory T cells with less‐differentiated phenotypes (CD27^+^CD57^−^) are associated with reduced CMV reactivation possibility in recipients after HSCT.^29^ In contrast, phenotypic analysis of CMV‐specific CTLs in 50 allografts showed that the high frequency of T_EMRA_ cells, when the number of T_EM_ is sufficient_,_ could decrease the risk of CMV reactivation.^30^ Furthermore, receiving manipulated grafts with CD45RA (naïve T cell) depletion leads to loss of T_EMRA_ cells and 3‐ to 5‐folds lower CMV‐specific immune response, compared to the CD62L‐depleted T cell‐enriched fractions.^31^ More researches are needed to find the principal memory T cell subsets that induce optimal antiviral immunity following adoptive therapy. Such studies may provide informative data about manipulation of graft cell composition and retaining protective T cells based on phenotypic composition of T‐cell populations.

### 3.3 T cell isolation strategies

CMV‐specific T cells for ACT could be generated by two main methods: direct isolation of CMV‐specific T cells and ex vivo expansion of virus‐specific T cells. The main CMV‐specific ACT studies are summarized in Table 1.

**TABLE 1:** Cytomegalovirus (CMV)‐specific Adoptive T cell therapy (ACT) clinical studies

Method of T cell manufacturing	Purpose	Infused cells	Source of T cell	Antiviral response
Direct isolation of Virus‐specific T cell 1. Short‐time ex vivo stimulation and Cytokine capture system (INF‐γ‐capture)				
Peggs et al (2010) (phase I–II)	Prophylactic^a^or Preemptive^a^	CMV‐specific CD4^+^ and CD8^+^ T cells	CMV seropositivedonors	• In prophylactic treatment, none of the patients required antiviral drugs within the next 6 months. • In preemptive treatment, 9 out of 11 patients required antiviral drugs. • CMV‐reactive CD4^+^ cells were detected in five out of six patients within 2 weeks of infusion. • CD8^+^ T cells were detected in seven patients within 2 weeks. • The patients who received the lowest dose of CMV‐specific T cells showed the slowest both CD4^+^ (3 weeks) and CD8^+^ (8 weeks) T cell immunity reconstitution.
Feuchtinger et al (2010)	Therapeutic for refractory CMV infection and CMV disease	CMV‐specific CD4^+^ and CD8^+^ T cells	CMV seropositive donors	• In 15 out of 18 cases (85%) CMV viremia cleared or the viral load (1 log) reduced significantly. • Only three patients did not response to adoptive pp65‐specific T cells transfer. • All cases showed in vivo expansion of CD4^+^ as well as CD8^+^ T cell.
Meij et al (2012) (phase I‐II)	Therapeutic for refractory CMV	CMV‐specific CD8^+^ T cells	CMV seropositive donors or autologous	• In all patients, CMV DNA load turned negative and CMV‐specific T cells detected in the PB. • CMV‐specific T cells in PB were CD8^+^ effector and memory T cells.
Mackinnon et al (2007)	Preemptive and prophylactic	CMV‐specific CD4^+^ and CD8^+^ T cells	CMVseropositive donors	• In 8 out of 23 patients who received cultured CMV‐specific T cells viral DNA cleared without antiviral drugs. • All patients (n = 7) showed in vivo expansion of CMV‐reactive T cells. • Viral titers decreased within 5 days. CMV‐reactive T cells represented a mean of 9.0% CD4^+^ cells and 7.3% CD8+ cells in 2–4 weeks.
Ingels et al (2020) (case report)	Therapeutic for multidrug resistant CMV infection	CMV‐specific CD4^+^ and CD8^+^ T cells	CMV seropositive donors	• CMV‐specific CD4^+^ T cells in the PB, expanded, and reached a frequency similar to the donor in 2 weeks after infusion and remained stable during the follow‐up period of 8 weeks. • CMVpp65‐specific CD8^+^ T even exceeded the frequencies found in healthy CMV seropositive individuals. • Reduction of CMV DNA copies in PB to <500 IU/mL
Direct isolation of virus‐specific T cell 2. Peptide‐MHC (pMHC) multimer				
Cobbold et al (2005)	Preemptive and therapeutic	CMV‐ specific CD8^+^ T cells	CMV seropositivedonors	• CTL response detected between 12 and 30 days after adoptive transfer • CMV viremia reduced in all cases and the infection cleared in 90% of patients. • CMV viral load reduced in one patient with refractory CMV infection
Schmitt et al (2010)	Therapeutic	CMV‐specific CD8^+^ T cells	CMV‐seropositivedonors	• CMV‐specific CD8+ T cells had an effector memory phenotype • The clearance of the CMV antigenemia was persistent. • Treatment with toxic antiviral drugs discontinued.
Neuenhahn et al (2017) (Phase I‐IIa)	Therapeutic for drug‐refractory CMV infection	CMV‐specific CD8^+^ T cells	CMV‐seropositivedonors or CMV‐seropositive Third‐party donors	In treated D^+^ patients: • CMV‐specific T cells detected in 2 to 3 weeks after transfer. • Treatment resulted in 62.5% complete and 25% partial responses. In treated D^−^ patients who received TPD‐derived CMV‐ specific T cells • CMV‐specific T cells were not detectable in 62.5% of patients. • Complete virus load response observed in 37.5% of patients. • T cell expansion triggered by a secondary viremic episode after adoptive transfer. • High HLA concordance between patient and TPD is an important requirement for successful virus‐specific ACT.
Ex vivo expansion of virus‐ specific T cells				
Riddell et al (1992)	Prophylactic	CMV‐specific CD8^+^ T cells	CMV‐seropositive donors	• CMV‐specific CD8^+^ T cell detected 48 hours after the first T cell infusion. • Immune responses increased after each cell infusion. • After the third cell infusion, lytic activity magnified in all recipients.
Walter et al (1995)	Prophylactic	CMV‐specific CD8^+^ T cells	CMV‐seropositivedonors	• Cytotoxic T cells specific for CMV reconstituted in all patients. • Transferred clones persisted for at least 12 weeks. • Cytotoxic‐T‐cells activity declined in patients deficient in CD4^+^ T‐helper cells specific for CMV. • CMV viremia and disease did not develop in any of the 14 patients.
Einsele et al (2002)	Therapeutic for drug‐refractory CMV infection	CMV‐specific CD4^+^ and CD8^+^ T cells	CMV‐seropositivedonors	• CMV viral load dropped despite antiviral drugs cessation in all patients. • Complete response detected in around 72% of patients. • Transient reductions in viral load detected in two out of seven patients who received an intensified immune suppression at the time of or after T‐cell therapy.
Peggs et al (2004)	Preemptive and therapeutic	CMV‐specific CD4^+^ and CD8^+^ T cells	CMV seropositive donor	• In eight patients (50%) viral DNA cleared without antiviral drugs. • CD8^+^ T cell numbers increased within 4 weeks in all cases. • A low incidence of late CMV reactivation was observed.
Micklethwaite et al (2007)	Prophylactic	CMV‐specific CD8^+^ T cells	CMV seropositive donor	• Specific T cells raised within the first 7 days after transferring in six out of nine recipients. • CMV reactivated in two recipients without pharmacotherapy or CMV disease development. • Specific T cells detected for the following 3 months.
Micklethwaite et al (2008)	Prophylactic	CMV‐specific CD4^+^ and CD8^+^ T cells	CMV seropositive donor	• All 12 patients demonstrated CMV‐specific immunity for at least one time point after ACT. • Low‐level DNAemia was detected in four patients after infusion.
Peggs et al (2009) (open‐label phase II)	Prophylactic, preemptive, and therapeutic	CMV‐specific CD4^+^ and CD8^+^ T cells	CMV seropositive donor	• In ACT prophylactic therapy, 30% of patients showed a primary CMV infection required antiviral therapy. • All 10 patients treated pre‐emptively required antiviral drugs due to the high viral titers. • CD8^+^ T cells number increased to the normal reference interval within 4 weeks in 28 patients. • CD4^+^ T cells count increased less rapidly. • The median pp65‐specific T cells number decreased by 3 months following transfer but were maintained at protective levels against uncontrolled viral replication.
Bao et al (2012)	Therapeutic for persistent or drug‐refractory CMV infection	CMV specific CD8^+^ T cell	CMV seropositive donor	• In five out of the seven new onset patients, CMV‐specific CTL activity detected within 4 to 6 weeks post infusion. • Among all seven patients with persistent CMV who received CMV‐specific CTL infusions, four patients developed specific cytotoxicity 4 weeks, and one patient developed specific cytotoxicity 6 weeks post‐infusion.
Blyth et al (2013) (phase II)	Prophylactic	CMV‐specific CD8^+^ T cells	CMV‐seropositive donors	• The incidence of CMV reactivation in the 50 patients treated with CTL was 26/50 (52%). In 9 out of 26 patients with CMV reactivation, antiviral medications were required.

a Prophylactic therapy is an approach to prevent CMV infection posttransplant. Preemptive therapy is preventive approach for CMV infection following viremia detection and before the onset of symptoms or tissue invasion.

#### 3.3.1 Direct isolation of virus‐specific T cells

This method is based on the direct isolation of donors' polyclonal CD4^+^ and CD8^+^ memory T cells and transfusion into the recipient. First, donors' blood leukocytes are collected by apheresis procedure; then donor PBMCs may specifically stimulate and activate with virus antigens (CMV lysate or immunodominant peptides such as pp65‐ derived peptides) for a short time (less than 24 hours). In the next step, virus‐specific T cells are isolated by two established methods: cytokine capture system and peptide‐MHC multimers. Finally, enriched T cells are administered to recipients.^32^

**1‐1‐3‐3‐ Cytokine capture system (CCS)** isolates specific T cells based on releasing specific cytokine after ex vivo stimulation for a short time. A common CCS‐based adoptive therapy is the purification of antigen‐specific CD4^+^ and CD8^+^ T cells secreting IFN‐γ.^33^ In this method, donors' activated T cells are labeled with anti‐CD45 antibody conjugated with anti‐IFN‐γ antibody, called catch reagent. Then, the caught cytokine‐secreting cells are subsequently labeled with a second anti‐IFN‐γ monoclonal antibody conjugated with super‐magnetic particles and enriched by magnetic cell sorting column.^16^, ^34^ The advantage of the cytokine capture method is its ability to isolate both CD4^+^‐ and CD8^+^‐specific T cells. Despite favorable results, this method has some requirements, such as CMV seropositive donor, high blood volume to obtain adequate CMV‐specific T cells, one or more potent virus‐specific stimulator for T cells activation, and good manufacturing practice (GMP) facilities to virus‐specific T cell isolation.

**2‐1‐3‐3‐ Peptide‐MHC multimer** has become a standard method for direct detection, phenotyping, enumeration, and isolation of antigen‐specific T cells within polyclonal T cell populations. This method is based on the specificity of peptide‐MHC (pMHC) recognition by TCR.^35^ To date, different types of pMHC complex format, including monomer, dimer, tetramer, pentamer, NTAmer, and dextramer, have become available for immunological applications.^36^ Clinical ACTs usually use tetramers streptavidin‐based platform, named streptamer, for pMHC multimers. The streptamer platform consists of four biotinylated synthetic peptide‐loaded recombinant MHC molecules binding to a magnetic bead‐conjugated streptavidin. In the first step, donors' PBMCs are labeled with CMV‐specific magnetic streptamer complex. Then, labeled cells are separated from other cells by a magnetic field. The purified T cells are eluted and released from the streptamer complex by adding biotin to the yield. The pMHC multimer technique is limited to specific peptides and particular MHCs. Currently, the GMP grade MHC class‐II‐peptide multimers are not available, and pMHC selection is limited to MHC class I‐peptide multimers and specific CD8^+^ T cell products.

#### 3.3.2 Ex vivo expansion of virus‐specific T cells

This method is based on the development of CMV‐specific polyclonal T cells for adoptive therapy. A donor's PBMCs or enriched T cells are co‐cultured with autologous virus‐infected fibroblasts or viral peptide‐pulsed dendritic cells (DCs) in an appropriate culture medium supplemented with inactivated human AB serum and cytokines (eg, recombinant human IL‐2). Subsequently, cells are restimulated and passaged weekly until reaching an optimal number of specific T cell clones.^20^ GMP grade gene‐modified APCs such as DCs nucleofected with viral antigen‐encoding DNA plasmid and DCs pulsed with overlapping multi‐epitope peptides have facilitated ex vivo expansion of specific T cells for clinical usage.^17^, ^37^, ^38^, ^39^, ^40^, ^41^ Moreover, generating CMV‐specific T cells from seronegative and cord blood donors has been provided by advancement in ex vivo expansion technologies. Nevertheless, ex vivo expansion of virus‐specific T cells is a long‐term process (4‐12 weeks), and it is not suitable in patients waiting for immediate medical treatment. This technique is limited by HLA restriction and expensive manufacturing procedure of GMP‐grade viral plasmid, peptide‐pulsed DC, and cell culture. Furthermore, ex vivo T cells overstimulation may be associated with overexpression of pro‐apoptotic molecules (such as Fas), downregulation of co‐stimulatory molecules (such as CD28) on T cells, and exhaustion of T cells.^42^

### 3.4 T cell qualification

Both ex vivo expanded‐ and direct isolated‐T cells are qualitatively and quantitatively evaluated prior to administration. Sterility tests are performed to monitor bacterial, fungal, viral, and mycoplasma contaminations of T cell products. Phenotypic and functional characteristics of T cells are evaluated with different laboratory tests. The frequency, phenotype, and specificity of T cells against CMV are usually assessed based on peptide‐MHC complex staining and cell surface/intracellular multicolor staining. The staining results are then analyzed by the flow cytometry method. The specificity of T cells could be analyzed with the enzyme‐linked immunospot (ELISPOT) test. Cytotoxicity assay is also used for the assessment of cytotoxic function mediated by T cell products. Mixed lymphocyte reaction and lymphoproliferation assay are performed to measure alloreactivity and clonal proliferation, respectively.^11^, ^17^, ^43^

## 4 CMV‐SPECIFIC ENGINEERED T CELLS

**FIGURE 1:**
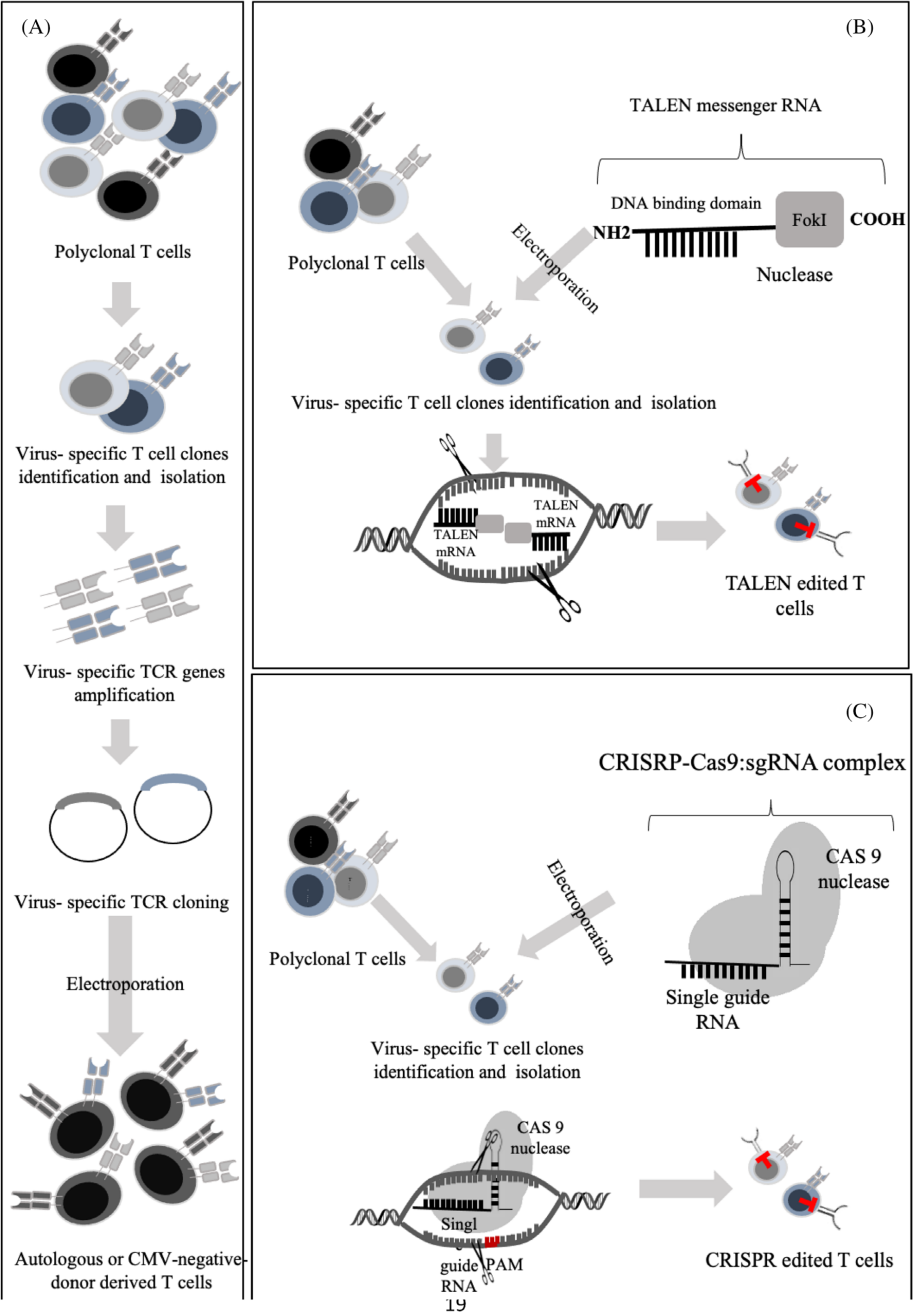
Virus‐specific engineered T cells. (A) Generation of TCR‐edited T cells: virus‐specific T cell isolation from polyclonal T cell population is performed via different methods, including co‐culturing with different HLA I‐ and II‐ restricted epitopes of virus specific Ag, cytokine capture system, and/or peptide‐MHC multimer. RNA from T cell clones is extracted, cDNA is synthesized, and TCR α and β chains are amplified via PCR and then analyzed. TCR α and β chains are separately inserted into viral vectors, amplified, and verified by DNA sequencing. Patient‐ or donor‐derived PBMCs are activated and transduced by viral vectors containing virus‐specific TCR chain genes to generate virus‐specific T cells. (B) Generation of drug‐resistant T cells (TALEN‐edited T cells): virus‐specific T cells are isolated and expanded from polyclonal T cell population. TALEN, as restriction enzyme, is engineered to cut specific DNA sequences of drug receptor (eg, glucocorticoid receptor). TALEN messenger RNA is transferred into expanded virus‐specific T cells by electroporation. Drug receptor‐targeting TALEN causes site‐specific double‐stranded DNA breaks and triggers natural DNA repair (nonhomologous end joining) that induces deletions of drug receptor gene in T cells. Finally, drug resistance is experimented in vitro and in vitro and functional capacity of TALEN‐modified T cells is confirmed. (C) CRISPR‐Cas9‐edited T cells: CRISPR‐Cas9, as an RNA‐programmable DNA targeting tool, could target specific genes and knock out drug receptors. CRISPR‐Cas9 induces site‐specific double‐stranded breaks in the target DNA in the presence of a short protospacer adjacent motif (PAM) flanking the target site. After the target site recognition, and following complementary base pairing between the synthetic guide RNA and target DNA, R‐loop is formed and cut into DNA strands. Then, Cas9 interacts with DNA that leads to conformational changes.^50^ Virus‐specific T cells are isolated from polyclonal T cell population and expanded. Specific drug receptor knocked‐out by CRISPR/Cas9 can be transferred into isolated virus‐specific T cells by electroporation. Knock‐out efficiency is evaluated via PCR amplification and DNA sequencing of PCR products

As mentioned before, HLA restriction and large blood volume requirement hinder the ACT application. Furthermore, patients who receive transplants from CMV‐negative donors are at higher risk of CMV reactivation because of lacking CMV‐specific T cells. Different strategies have been developed to tackle these problems. In some studies, seronegative donors were vaccinated and their CMV‐specific T cells were isolated and expanded ex vivo to resolve infection.^44^, ^45^ In addition, establishing a third‐party donor‐derived CMV‐specific T cell bank provides off‐the‐shelf ACT in patients who received transplants from CMV‐negative donors or need immediate clinical care.^46^, ^47^ These approaches are restricted to HLA compatibility between donor and recipient. New strategies are needed to develop HLA‐unrestricted methods. Genetic engineering can address these problems and make ACT widely applicable. Advanced genetic engineering makes it possible to rapidly generate transgenic virus‐specific T cells either from the patient (autologous) or donor with seronegative status. Engineered T cells can be used adoptively to redirect patient virus‐specific T cell response and improve ACT. Moreover, T cells can be genetically modified to recognize antigens in an MHC‐independent manner and to preserve their protective functions upon concurrent treatments. TCR‐transgenic T cells^48^ and chimeric antigen receptor (CAR) T cells^49^ have been investigated as engineering‐based T cells to treat infectious diseases (Figure 1).

### 4.1 CMV‐specific TCR‐transgenic T cell

TCR is a heterodimer transmembrane protein consisting of either alpha/beta or gamma/delta chains, which recognizes specific antigens in the context of peptide‐MHC complex.^51^ TCR‐engineered T cells are antigen‐specific and reproducible cells produced based on genetic modification manner. Transgenic TCR‐based ACT has shown promising results in some solid and hematologic malignancies as well as viral‐associated malignancies such as sarcoma, melanoma, multiple myeloma, acute myeloid leukemia, and human papillomavirus (HPV)‐associated cancers.^52^, ^53^, ^54^, ^55^

Several studies have demonstrated that TCR‐engineered T cells could also be implemented to redirect T cell responses against viral infections. The possibility of engineered T cell production was examined to target the human immunodeficiency virus (HIV),^56^ HPV,^57^ hepatitis B virus (HBV),^58^ hepatitis C virus (HCV),^59^ and Epstein‐Barr virus (EBV).^60^

This strategy relies on gene cloning methods. Basically, virus‐specific T cell clones could be separated from healthy immunized donors or generated by stimulating nonspecific lymphocytes by virus‐specific epitopes restricted to various HLAs. After isolating TCR genes from specific T cell clones, cDNA is synthesized, the specificity of alpha and beta chain genes is determined and verified by DNA sequencing, and finally, alpha and beta chain genes are amplified by PCR. Then, TCR alpha and beta chain genes are cloned into an expressing viral vector, especially oncoretroviral and lentiviral vectors, to produce particles containing specific TCR genes. Afterward, cloned TCR genes are transduced to previously activated or nonactivated target cells (normal peripheral blood lymphocyte). TCR‐transfected cells are then restimulated with specific antigens and functionally analyzed. Finally, TCR‐transfected cells are enriched and expanded for clinical applications.^61^

Schub et al revealed the feasibility of transferring CMV‐specific TCR into CMV negative donor‐derived primary T cells. These engineered lymphocytes showed potential effector function such as releasing effector cytokines (IL‐2 and IFN‐γ), substantial cytotoxic activity, as well as enrichment and expansion against endogenously processed CMV antigen pp65. Moreover, they indicated that CMV‐TCR‐transgenic T cells could maintain their memory phenotype after repeated ex vivo antigen exposure.^62^ Although conventional TCR‐based ACT is HLA‐restricted, identifying CMV‐specific TCR chain sequences that are restricted to the highly frequent HLA alleles found in the population can provide a TCR repertoire to mitigate HLA restriction of ACT.^63^, ^64^, ^65^

#### 4.1.1 Soluble high‐affinity TCR‐engineered T cells

A suitable engineered T cell for adoptive transferring should express efficient TCR in sufficient numbers. Based on these facts, expression, stability, and affinity of engineered T cell could be enhanced by using some methods like removing additional unstable mRNA motifs, synthetizing murinized TCR, formatting single‐chain TCR, and employment of phage display techniques.^57^, ^66^, ^67^

Regarding TCRs' biochemical and structural similarities to antibodies, they have the potential capacity in specific antigen recognition and can be engineered to act as antibody‐like reagents. These TCRs are expressed on virus‐infected cells and could be used for viral infection diagnosis and treatment. Various studies declared that soluble TCRs could inhibit HIV replication, control immune escape and shift CD8^+^ T cell responses to eliminate infected CD4^+^ T cells after HIV reactivation.^68^, ^69^

Wagner et al established a new mammalian cell‐based platform for engineering CMV‐specific TCRs with higher receptor affinity and stability in a soluble format. They improved the affinity of TCRs by introducing an interchain disulfide bond and mutation in the CDR3 domain of alpha and beta chains. They also fused the TCR antigen‐binding domain into the constant domains of an antibody to improve protein expression and stability.^70^ The soluble TCR‐antibody fusion protein with increased affinity and high specificity could bind to CMV‐infected cells and predispose them to immune clearance. Moreover, they can replace the CMV antigenemia assay to diagnose infection status in transplant recipients or monitor CMV presentation after vaccination.^70^, ^71^

#### 4.1.2 Drug‐resistant TCR‐engineered T cells

Although ACT could restore protective immune response in transplant recipients, ongoing concurrent immunosuppressive treatments can limit its efficacy. Most of the CMV‐specific ACT studies have analyzed the transplanted patients without GVHD. Nevertheless, GVHD patients receiving systemic corticosteroid therapy might be at a high risk of CMV reactivation and treatment resistance. Corticosteroids are the first‐line treatment for GVHD and could suppress the immune system.^72^ As a corticosteroid drug, dexamethasone inhibits T cell activation and proliferation but does not affect their cytotoxicity. However, some other immunosuppressive drugs such as cyclophosphamide and methotrexate could inhibit T cell activation and cytotoxic capacity.^73^ Therefore, engineering T cells with resistance capacity against immunosuppressive drugs can make ACT applicable for a wide range of patients. Different tools are now available for gene editing, including transcription activator‐like effector nucleases (TALENs)^74^ and clustered regularly interspaced short palindromic repeats/Cas9 (CRISPR/Cas9).^75^ Menger et al engineered TALEN‐modified CMV‐specific CD8^+^ T cells that maintained cytotoxic activity against CMV peptide‐pulsed (pp65) targets in the presence of dexamethasone. They inactivated glucocorticoids receptor (GR) in CMV‐specific T cells using TALEN messenger RNA targeting GR. They also confirmed T cell resistance to corticoids in a xenogeneic GVHD mouse model.^76^ Kaeuferle et al generated glucocorticoid‐resistant CMV‐specific T cells by knocking out the endogenous GR via CRISPR/Cas9 gene‐editing technology. They demonstrated that CRISPR/Cas9 engineering did not alter T cell phenotype, cytokine release, and cytotoxic capacity. In addition, GR‐knocked out T cells revealed higher expansion capacity than GR wild‐type T cells that could be enriched during dexamethasone treatment. The risks of uncontrolled activation and resistance to other immunosuppressive compounds are the main concerns in corticoid‐resistant T cell clinical application. The safety of GR‐knocked out T cells is confirmed via suppressing these cells by other immunosuppressive compounds like calcineurin‐inhibitors.^77^ The first clinical trial (phase I) on patients with refractory cancers confirmed the safety and feasibility of infusing CRISPR‐Cas9 gene editing T cells^78^ (Figure 1).

### 4.2 CMV‐specific CAR T cells

CAR is an engineered fusion protein comprised of an extracellular single‐chain variable fragment (scFV) antigen‐binding domain, spacer domain, a transmembrane domain, and intracellular domains, including CD3 zeta chain‐derived signaling domain and costimulatory domains such as CD28 and 4‐1BB.^79^ Promising results obtained from CAR T cell therapy for cancer has motivated researchers to design CAR for infectious disease.^80^ CAR technology enables autologous virus‐specific CTL development independent of HLA restriction.^81^ The first virus‐specific CAR T cell was generated to cure HIV infection.^82^, ^83^ Currently, CAR T cells could target HBV,^84^ HCV,^85^ and CMV.^86^

The first CMV‐specific engineered CAR T cell was designed against glycoprotein B (gB), which is expressed on the surface of infected cells during the early and late CMV replication phase. It was generated via fusing extracellular gB scFv to an immunoglobulin hinge region and intracellular signaling domains of the CD28 and CD3.^86^ Although gB‐CAR‐expressing T cells activated and released cytokines and cytotoxic granules in a co‐culture system, they were not able to efficiently induce apoptosis or lysis in CMV infected cells.^86^, ^87^ A further study proved that gB‐CAR T cells could mediate the inhibition of CMV replication, independent of cytotoxicity and mainly via IFN‐γ and TNF secretion.^88^ It is suggested that viral anti‐apoptotic factors, which inhibit apoptosis in infected cells, might abrogate CAR T cell cytotoxicity.^86^, ^87^ Although gB‐CAR T cells did not induce cytotoxic effect, Ali et al generated anti‐CMV neutralizing antibody‐based CARs that were able to kill infected cells by cytolysis.^89^

**FIGURE 2:**
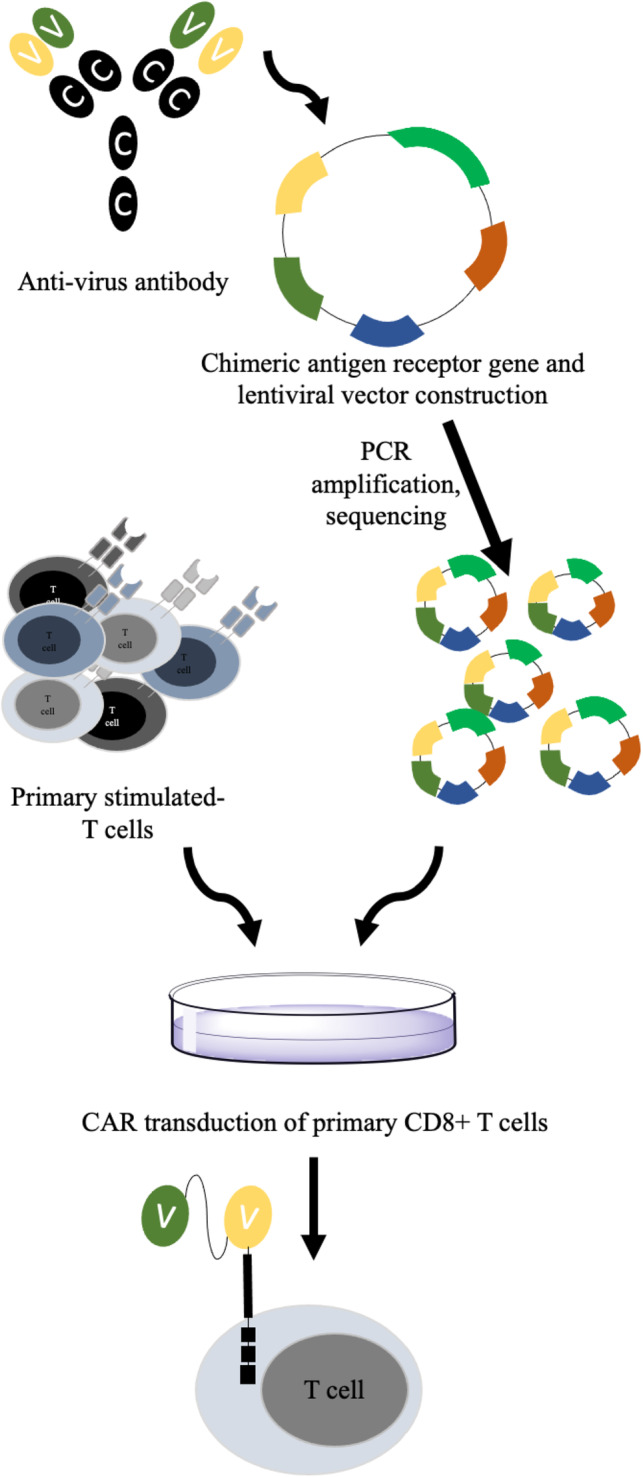
Virus‐specific CAR T cells. To synthetize scFv fragment of chimeric antigen receptor (CAR), neutralizing antibodies against virus is produced in hybridoma cells, and their RNA is extracted to synthesis cDNA. Heavy and light chains variable regions are amplified by PCR, and PCR products are confirmed by DNA sequencing. PCR products are inserted into viral vectors containing an Ig‐based hinge region, TCR transmembrane domain, co‐signaling domain, and intracellular signaling domains (CD28 and CD3). The cloned viral vectors containing CAR sequences are amplified. Primary CD8^+^ T cells are isolated from healthy donor, and stimulated in vitro. Viral vectors are delivered to stimulated primary T cells and cultured in appropriate cell culture medium. Enriched CAR T cells are then confirmed by functional testing experiments

It is revealed that CMV encodes Fc binding receptor that expresses on the infected cell surface and interferes with antibody‐mediated immune response.^90^ Therefore, CMV‐encoded FcRs represent an attractive opportunity for CAR T cell designing. Proff et al established a CAR T cell containing mutant IgG1‐ and IgG4‐derived CH2‐CH3 spacer domains, which could recognize CMV‐encoded FcRs without interacting with endogenous FcRs. It seems that the efficacy of CMV‐specific CAR T cells can be improved by bispecific antibody constructs.^88^ New strategies should be considered to design more efficient CARs with improved protective functions that could decrease the viral immune escape (Figure 2).

## 5 CONCLUSION

Conditioning regimens and immune suppressive drugs used following the HSCT process could lead to impairments in the immune system and may increase the risk of infection in recipients. CMV‐specific T cells are essential to control CMV infection/reactivation post‐HSCT. Therefore, rapid reconstitution of T cell immunity against CMV may improve HSCT outcomes. Adaptive transferring of CMV‐specific T cells from seropositive donors into recipients could reestablish T cell responses in a short time. The emergence of rapid ex vivo expanding virus‐specific T cells and new T cell isolation methods, along with the establishment of third‐party banks, have increased ACT applicability. Nevertheless, several hurdles such as restriction of HLA between donor and recipient, the need for optimization of infused T cell subsets, and sensitivity of T cells to commonly used immunosuppressive drugs have highly restrained ACT applications. Manufacturing TCR‐engineered T cells and CAR T cells are new promising approaches to achieve immunosuppressive‐resistant HLA‐independent T cells. To date, there have been limited reports on CMV‐specific engineered T cell applications in clinical settings. Further studies are required to prove the efficacy and safety of these products.

## FUNDING

This research did not receive any specific grant from funding agencies in the public, commercial, or not‐for‐profit sectors.

## CONFLICT OF INTEREST

The authors declare no conflict of interest.

## AUTHOR CONTRIBUTION

Conceptualization and Supervision: Abbas Hajifathali, Mohsen Hamidpour.

Writing – Review & Editing: Mahshid Mehdizadeh.

Writing –Original Draft: Samira Karami, Haniyeh Ghaffari Nazari, Ghazaleh Sankanian.

All authors revised the manuscript and approved the final paper. Conception or design of the work.

## ETHICS STATEMENT

Not applicable.

## Data Availability

Data sharing is not applicable to this article as no new data were created or analyzed in this study.
